# Integrating gut microbiota into multidisciplinary perspectives on diabetic neuropathy

**DOI:** 10.3389/fendo.2025.1710868

**Published:** 2025-11-03

**Authors:** Maksym Horiachok, Kateryna Potapova, Taras Ivanykovych, Viktoria Yerokhovych, Yeva Ilkiv, Larysa Sokolova

**Affiliations:** ^1^ Global Medical Knowledge Alliance, Boston, MA, United States; ^2^ Department of Neurology, Bogomolets National Medical University, Kyiv, Ukraine; ^3^ Danylo Halytsky Lviv National Medical University, Lviv, Ukraine; ^4^ Endocrinology Department, Bogomolets National Medical University, Kyiv, Ukraine

**Keywords:** gut microbiota, diabetic neuropathy, gut-brain-peripheral nerve axis, metabolomics, microbiota-targeted management

## Abstract

Diabetic neuropathy (DN) is one of the most common and debilitating complications of diabetes mellitus, yet its precise pathogenesis remains incomplete. Emerging evidence highlights the gut microbiome as a key factor linking metabolic dysfunction, immune activation, and neuronal damage. Even minor dysbiosis may interfere with microbial metabolite balance and disrupt intestinal integrity, leading to local and, consequently, systemic inflammation, which in turn drives altered pain response via the gut-brain-immune axis. Recent clinical and preclinical data show that reduced short-chain fatty acid availability, altered bile acid and tryptophan metabolism, let alone expansion of pro-inflammatory species collaboratively contribute to DN onset and progression. Moreover, advances in metagenomics and metabolomics reveal reproducible microbiome-derived biomarkers that could predict neuropathy risk and pain phenotypes independent of glycemic control, supporting the microbiome as both a mechanistic driver and a measurable potential diagnostic tool. In the context of management, microbiota-affected interventions, such as probiotics, synbiotics, omega-3 supplementation, and fecal microbiota transplantation, show early promise in alleviating symptoms and improving nerve function. This mini-review synthesizes current evidence on the microbiome’s role in DN, emphasizing its dual potential as a biomarker for early diagnosis and a therapeutic target for precision microbiome-based interventions.

## 1 Introduction

Diabetic neuropathy (DN) is among the most frequent complications of both types 1 and 2 diabetes mellitus (T1DM and T2DM), affecting up to 50% of diabetic patients (1). The current prevalence of diabetes is estimated to be 537 million adults across all age groups and continents. Moreover, the number has been increasing and is projected to rise to 783 million by 2045, even more terrifying that by 2050 this number could reach up to 1.31 billion (2). Reported prevalence of diabetic neuropathy differs among various age groups, cultural and geographical factors, diverse diagnostic tools, and considering glucose state peculiarities. For instance, the MONICA/KORA study has shown that the overall prevalence of neuropathic pain was 13.3% among people with diabetes, 8.7% with glucose intolerance, 4.2% in those with impaired fasting glucose level, and only 1.2% of normoglycemic individuals (3). Additionally, different diagnostic tools yield relatively diverge ranges, estimating at 11-13% of DN among individuals with T1DM diagnosed with the Michigan Neuropathy Screening Instrument (MNSIQ) (4, 5), in opposite to 28% when diagnosed clinically measuring vibration perception thresholds (6, 7). Several classifications of the diverse syndromes affecting the peripheral nervous system in diabetes have been proposed in recent years. Here, we presented the adapted classification from originally described by Thomas (**Table 1**) (8).

**Table 1:** Classification of diabetic neuropathy.

Type of neuropathy	Subtypes
Generalized symmetric polyneuropathies	Acute sensory
	Chronic sensorimotor
	Autonomic
Focal and multifocal neuropathies	Cranial
	Truncal
	Focal limb
	Proximal motor (amyotrophy)
	Coexisting CIDP

CIDP = chronic inflammatory demyelinating polyneuropathy.

The precise cause of DN remains incompletely defined. While it has been proposed to have several causal mechanisms, such as metabolic, neurovascular, and autoimmune (9), a large number of studies have found a link between the diversity of gut microbiome and the development of DN (10). The major factors that contribute to the development and progression of DN are the duration of diabetes, status of glycemic control, age, obesity, dyslipidemia, insulin resistance, lifestyle habits, cardiovascular health, let alone chronic inflammation and genetic predispositions (2, 11, 12). Beyond these established and well-studied risk factors, accumulating evidence suggests the role of the gut in modulating glucose homeostasis and metabolic balance, which is furthermore deeply implicated in the pathogenesis of DN (13–17). Despite this understanding, the treatment efficacy for DN is often unsatisfactory; common pharmacological therapies include antidepressants, anticonvulsants, and analgesics, yet these treatments are frequently limited in effectiveness and may have adverse side effects (18). At the same time, research papers in other neurological disorders confirm that dysbiosis can actively shape disease trajectories. For example, a recent Ukrainian study of multiple sclerosis (MS) patients found that gut microbiota in Ukrainian MS patients differs significantly from healthy controls, with increased abundance of pro-inflammatory *Proteobacteria* and decreased levels of *Bacteroidetes* and *Firmicutes*. These findings highlight the immunomodulatory potential of the microbiome in CNS disorders and underscore the relevance across neuroinflammatory and neurodegenerative conditions, including DN (19). It has been found that intestinal microflora imbalance contributes to neuroinflammation, mitochondrial dysfunction, and oxidative stress — key factors in the pathogenesis of DN (20).

Given the multifactorial and systemic nature of DN, a multidisciplinary approach becomes inevitable. Effective management of such complexity requires a unique and coordinated effort among various specialties, including endocrinologists, neurologists, nutrition specialists, microbiome researchers, and lifestyle intervention specialists. This collaboration could not only enhance accuracy in diagnostic measurement and conclusions but also improve the development of modern therapeutic options, such as microbiota-targeted interventions. This perspective addresses the need to view DN not purely as a neurological complication of diabetes, but as a complex interplay between metabolic, vascular, immune, and microbial factors.

## 2 Gut-brain axis and immune response

**Figure 1:**
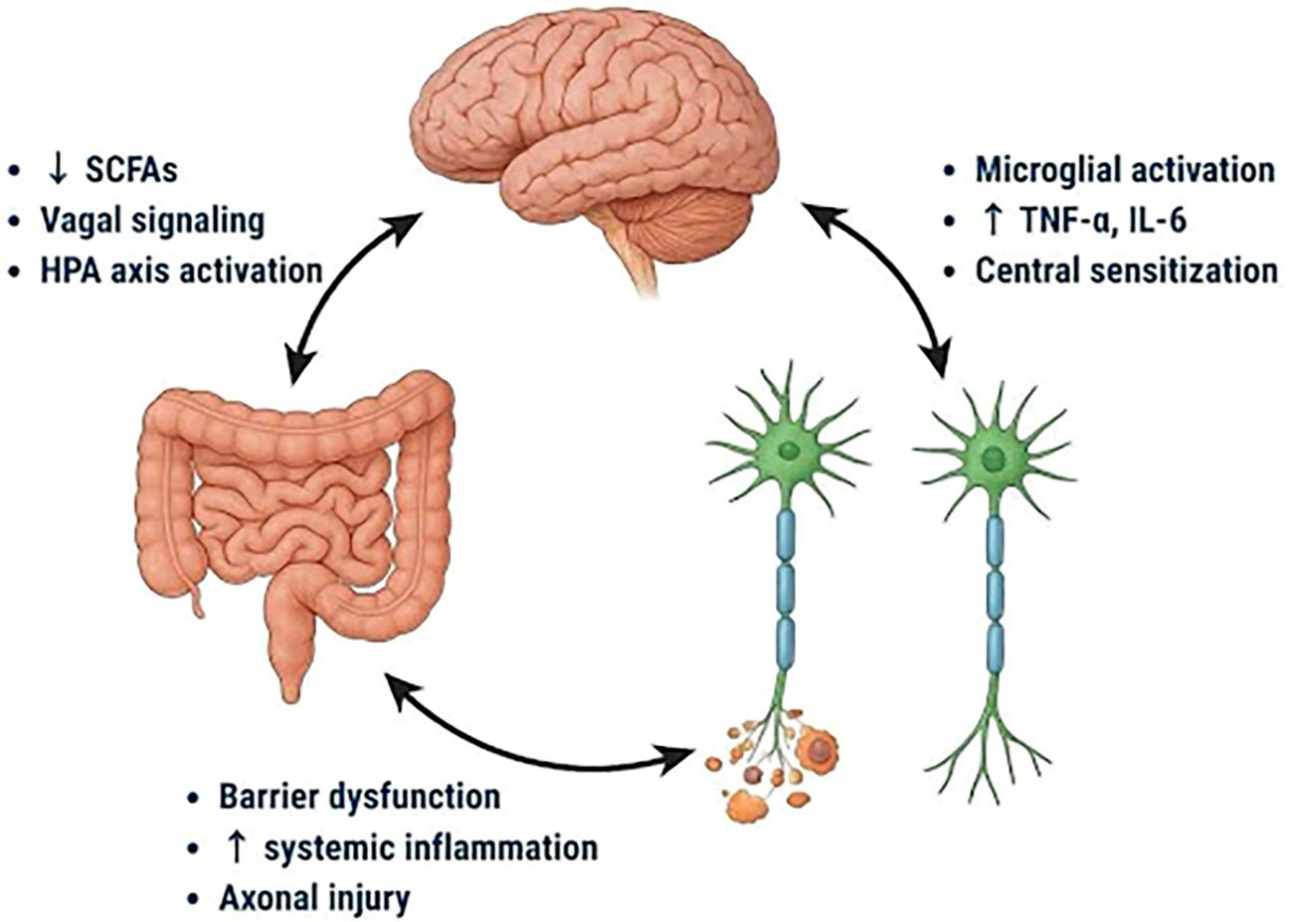
Bidirectional interactions between the gut, brain, and peripheral nerves in the pathogenesis of diabetic neuropathy. Microbiota-derived factors such as reduced short-chain fatty acids (SCFAs), barrier dysfunction, and systemic inflammation influence vagal signaling and hypothalamic-pituitary-adrenal (HPA) axis activation, promoting microglial activation, elevated tumor necrosis factor-alpha (TNF-α) and interleukin-6 (IL-6) levels, and central sensitization.

The relationship between the microbiota and diabetic neuropathy now focusses on the gut-brain-peripheral nerves (GB-PNA) axis. Based on recent research, intestinal dysbiosis induces systemic endotoxemia and disruption of the intestinal barrier, that consequently stimulates pattern recognition receptors (like TLR4) and kicks off a pro-inflammatory cascade involving cytokines like TNF-α and IL-6 (**Figure 1**). These inflammatory signals spread via immunological and neurological pathways, leading peripheral nerve axonal injury, glial activation, and mitochondrial dysfunction (21).

Notably, studies using Mendelian randomization offer proof for a causal relationship between specific gut taxa and the phenotype, and animal models have shown that inflammatory caused by the microbiota affects both central and peripheral sensitization pathways (22).

In addition, an increase in enterobacteria and a decrease in butyrate producers impair immune tolerance by increasing TNF-α and IL-6 signaling, which are associated with the progression of DN (21, 23, 24).

Microbial metabolites, particularly short-chain fatty acids (SCFAs), are required for maintaining epithelial and neuronal homeostasis, but reduced SCFA availability correlates with the severity of neuropathic pain (22, 25, 26). Preclinical studies show that dysbiosis modulates vagal signaling, hypothalamic-pituitary-adrenal activity, and microglial activation, which together enhance neuroinflammation (27–29).

Recent data also suggests that immune activation derived from the gut modifies mitochondrial function and oxidative stress in peripheral nerves, increasing axonal injury (19, 23, 28). Disruption in microbial metabolites, such as bile acids and tryptophan derivatives, has been found to affect the responsiveness of sensory neurons and pain processing pathways (27, 28). Additionally, genetic and causal studies indicate a two-way relationship, where both the microbiota composition and the body’s immune response aggravate neuropathic pathology (22, 25, 26, 30). Clinical data support these mechanisms, as patients with DN have different microbiome-immune characteristics compared to diabetics without neuropathy (30, 31).

## 3 Microbiome as a potent marker for DN

The latest developments in systems biology show that the microbiome can give us clinically important biomarkers for DN. As compared to mechanistic studies of the gut-brain-immune axis, biomarker research focuses on measurable microbial signs that have diagnostic and prognostic value. High-accuracy metagenomics and metabolomics have identified replicable taxonomic and functional changes in patients with DN, including loss of microbial biodiversity and overrepresentation of opportunistic taxa such as *Enterobacteriaceae* and *Ruminococcus gnavus* (32–34).

The metabolomic fingerprint adds to these findings, identifying promising biomarkers such as circulating form, altered indole derivatives, and distinct bile acid profiles that correlate with neuropathy severity and pain phenotypes (35–37). Importantly, these microbial metabolites can be detected in serum or feces, allowing for the development of non-invasive biomarkers.

Long-term studies strengthen their prognostic power, showing that microbiome characteristics measured before the onset of neuropathy can predict future nerve damage and pain regardless of glycemic control (26, 38). The incorporation of multiomics even more improves diagnostic accuracy: combined microbial, metabolic, and transcriptomic characteristics of the host are superior to single-omics methods in identifying patients at risk for DN (21, 25).

To illustrate these associations, **Table 2** summarizes key microbial alterations recently identified across different types of DN and experimental models, highlighting microbial link to greater or fewer risk of disease development.

**Table 2:** Microorganism alterations associated with different types of DN and their potential risk contribution.

Author	DN type	Microorganisms increased	Microorganisms decreased	Notes on Risk/Mechanism
Wang et al., 2020 (10)	Peripheral neuropathy (Type 2 DM, human study)	*Firmicutes*, *Proteobacteria*, *Escherichia-Shigella*, *Megamonas*	*Bacteroidetes*, *Faecalibacterium*	Higher *Proteobacteria* linked to endotoxemia and inflammation; reduced *Faecalibacterium* lowers SCFA production.
Jia et al., 2025 (31)	Painful diabetic neuropathy (rat model)	Pro-inflammatory colonic flora, ↑ TNF-α/IL-1β producing taxa	Butyrate producers	Altered microbial composition associated with colonic mucosal injury, systemic inflammation, and neuropathic pain behavior.
Xu et al., 2024 (39)	Diabetic peripheral neuropathy (MR study, genetic evidence)	*Ruminococcus 2*, *Clostridiales vadinBB60 group*	*Prevotella 9*, *Bacteroides*	Certain taxa genetically predicted to ↑ risk (pro-inflammatory), others (SCFA-producers) ↓ risk.
Jiang et al., 2025 (40)	Experimental DN (rat, probiotic intervention)	Pathogenic gut flora suppressed by probiotics	Restoration of *Lactobacillus*/*Bifidobacteria* balance	Probiotics reversed neuropathic pain and restored barrier function via inhibition of TLR4/MyD88/NF-κB pathway.
Shabani et al., 2023 (41)	Diabetic neuropathy (oxidative stress model, rats)	Opportunistic taxa associated with oxidative stress	Protective commensals enhanced by probiotics	Probiotics reduced oxidative damage, ↑ antioxidant enzymes, and alleviated DN symptoms.

In addition to bacteria, new data point to the diagnostic potential of the gut microbiome and virome, which may provide additional diagnostic markers for neuropathy subtypes (23). Approaches to causal inference, including Mendelian randomization, add reliability, confirming that specific microbial species and metabolites are not only associated with but also likely to be the cause of DN pathogenesis (33, 34).

Altogether, these results highlight that the microbiome is not only a mechanistic factor but also a dynamic and quantitatively measurable biomarker platform that can be applied for early diagnosis, risk stratification, and personalized monitoring of diabetic neuropathy.

## 4 Microbiota therapy as an emerging therapeutic approach

Preclinical data confirm the role of the gut microbiome as an early indicator of DN. In mice with streptozotocin-induced diabetic peripheral neuropathy, administration of the probiotic *Lacticaseibacillus rhamnosus* TR08 significantly relieved pain symptoms, improved sciatic nerve structure, and restored neurotransmitter and neurotrophic factor levels. Significantly, this was associated with improved gut barrier function and reduced systemic inflammation, as evidenced by decreased levels of IL-6, TNF-α, and LPS. These results emphasize that modulation of the gut microbiota can directly impact both central and peripheral components involved in DN pathology, strengthening its significance not only as a biomarker but also as a causal factor in disease development (38) .

While current treatment options for DN are primarily directed toward symptomatic management, such as glycemic control, analgesics, anticonvulsants, they have shown only limited success in halting neuropathy progression or reversing neuronal degeneration. This therapeutic gap has prompted increasing interest in novel disease-modifying methods. Among them, the gut microbiota has emerged as a potential target.

The gut-brain-axis has expanded to gut-brain-peripheral nerve axis and is now considered as a critical regulator of immune and metabolic homeostasis. While numerous studies hypothesize its importance, growing evidence suggests disruption directly affects the development and drives progression of DN. Thus, therapeutic strategies aimed at modulating the gut microbiota offer a unique opportunity not only to alleviate symptoms but also modify disease trajectory.

As of August 2025, several promising approaches have been represented, one of which is fecal microbiota transplant (FMT) (**Table 3**). A randomized, double-blind, and placebo-controlled trial demonstrated that through modulation of the composition and function of gut microbiota via FMT, neuropathic symptoms can be alleviated, and moreover, nerve function can be improved in patients with distal symmetric polyneuropathy (DSPN) (42). Another emerging potent therapeutic player has been considered in probiotic supplementation. In the randomized, double-blind, placebo-controlled trials has been shown probiotic supplementation for 12 weeks among patients with diabetic foot ulcer had a beneficial effect on glycemic control, total cholesterol, high-sensitivity C-reactive protein, plasma nitric oxide, and total antioxidant capacity (43). Similar conclusions have been drawn from meta-analysis of randomized controlled trials, suggesting that probiotic and synbiotic supplementation may help to improve biomarkers of inflammation and oxidative stress in diabetic patients (44).

**Table 3:** Summary of clinical studies on the effect of gut microbiota modulation for DN.

Author	Intervention	Research type and duration	Main results
Yang et al., 2023 (42)	Fecal microbiota transplantation	Randomized, double-blind, placebo-controlled trial (n=22)	Modulation of gut microbiota alleviated neuropathic symptoms and improved nerve function in patients with DSPN
Mohseni et al., 2018 (43)	Probiotic supplementation	Randomized, double-blind, placebo-controlled trial; 12 weeks (n=60)	Improved glycemic control, cholesterol, hs-CRP, plasma nitric oxide, and antioxidant capacity in patients with DFU
Zheng et al., 2019 (44)	Probiotic and synbiotic supplementation	Meta-analysis of randomized controlled trials	Improved biomarkers of inflammation and oxidative stress in diabetic patients
Sabico et al., 2019 (45)	*Bifidobacteria* and *Lactobacillus* supplementation	Single-center, double-blind, randomized, placebo-controlled clinical trial; 6 months (n=30)	Decrease in levels of endotoxin by 70%. Decrease in levels of glucose (38%), insulin (38%), HOMA-IR (64%)
Lewis et al., 2017 (46)	Omega-3 supplementation	Single-arm, open-label pilot trial; 12 months (n=40)	↑ Corneal nerve fiber length by 29% in T1DM patients; first proof-of-principle that therapy may reverse damage
Niimi & Sango, 2024 (47)	*Probiotics (L. rhamnosus TR08)*	Preclinical study in diabetic mice (STZ-induced)	↓ pain behavior, ↑ sciatic nerve structure, ↑ neurotransmitters and BDNF, ↓ IL-6/TNF-α/LPS; improved intestinal barrier integrity
Feng et al., 2022 (47)	Probiotic supplementation (*Lactobacillus rhamnosus TR08*)	Preclinical study in high-fat diet-induced dyslipidemic mice	TR08 improved gut microbiota composition, ↑ SCFA production, ↓ pro-inflammatory cytokines (IL-2, IFN-γ), and reduced vascular inflammation.

DSPN = distal symmetric polyneuropathy, hs-CRP = high-sensitivity C-reactive protein, DFU = diabetic foot ulcer, HOMA-IR = Homeostatic Model Assessment of Insulin Resistance, T1DM = type 1 diabetes mellitus, SZT-induced = streptozotocin-induced.

↑ = Increased.

↓ = Decreased.

Additionally, modulation of the gut microbiota by administration of *Bifidobacteria* and *Lactobacillus* or fecal transplantation has been shown to improve insulin resistance (45). The omega-3 supplementation has also shown promise. In a single-arm, open-label pilot trial, 12 months of omega-3 supplementation was associated with an increase in corneal nerve fiber length by 29% in patients with T1DM. While this study lacks a proven causality, the trial is the first to provide proof-of-principle data that a targeted interventional therapy can stop and reverse small fiber damage (46).

Beyond clinical research, recent patent analysis has highlighted the need to explore the benefits of probiotics and clinical use for patients with DN. A patent prospection gathered retrospective data from 48 inventions between 2009 to 2022 and showed a peak in patent filling in 2020, whereas Asian countries contributed to more than 50% of those inventions. The effects, such as reduction of pro-inflammatory mediators and hypoglycemic control, have been shown to generate mostly from two species *Bifidobacteria* and *Lactobacillus* (48). These findings underscore the substantial academic interest in probiotic supplementation, as well as growing commercial frontier in DN treatment.

## 5 Discussion

This mini review presents new findings that the gut microbiome contributes to the onset and progression of DN as a mechanical factor and as a biomarker platform. Quantitative findings indicate that up to 50% of patients with diabetes experience DN, with 13.3% of diabetics reporting neuropathic pain versus 1.2% of patients with normal blood glucose levels. Metagenomic and metabolomic studies demonstrate consistent loss of microbial biodiversity, reduced production of SCFA, and alterations in bile acid/tryptophan metabolism, along with an excessive representation of opportunistic species such as *Enterobacteriaceae* and *Ruminococcus gnavus*. Significantly, these microbial changes not only correspond with neuropathy severity but may also suggest future neuropathy risk independent of glycemic control, emphasizing their diagnostic and therapeutic potential.

Existing studies align in associating dysbiosis with systemic inflammation, mitochondrial dysfunction, and axonal injury via the gut-brain-immune axis. Clinical investigations reveal a reduction in SCFA availability and alterations in bile acid profiles among patients suffering from DN, alongside heightened pro-inflammatory cytokine activity. While certain studies emphasize taxonomic alterations, others pinpoint functional metabolic disruptions as significant factors in DN pathology. This inconsistency may stem from variations in methodology, dietary influences, or the diversity of patient populations. Furthermore, research, including Mendelian randomization, reinforces the notion that the gut microbiota is not merely correlated with DN pathogenesis but also plays an active role in its progression. Nonetheless, there remains debate over the specificity of microbial markers and the most dependable taxonomic classification (phylum versus genus versus species) for clinical use.

The combination of preclinical mechanistic studies with multi-omics research and early-stage interventional tests, which ensures a comprehensive and translational view, is what makes this combination so strong. To date, results are limited by small sample sizes, inconsistent methodologies for diagnosing DN, and variations in microbiome assessment techniques. Identification of reliable biomarkers is also complicated by the dynamic nature of the microbiota, which is context-dependent and influenced by factors such as age, diet, geographic location, and specific intestinal site. However, the capacity to replicate certain taxonomic and metabolic characteristics across different studies suggests that these findings are sufficiently valid to serve as a basis for further translational research.

The recognition of the importance of the gut microbiome in DN has major clinical implications. Innovative therapeutic biomarkers and treatment approaches, such as probiotics, synbiotics, omega-3 supplements, and fecal microbiota transplantation, provide opportunities not only to relieve neuropathic pain but also to modify disease progression. Significantly, these findings highlight the need for a joint, multidisciplinary strategy: neurologists, endocrinologists, diabetologists, and gastroenterologists must collaborate to include microbiome-focused diagnosis and treatment in patient care. This multidisciplinary collaboration is especially relevant considering the variability of DN presentations and the interactions between metabolic, neurological, and gastrointestinal elements. Consequently, upcoming clinical trials should be structured with multidisciplinary teams to guarantee a careful evaluation and management approach.

Several disadvantages are brought up in this review. Most of the referenced clinical studies have limited follow-up periods and small cohorts. Studies apply various microbiota analysis techniques and criteria for diagnosis for diabetic neuropathy. Additionally, the gut microbiota’s composition is extremely variable and depends on a few variables, including geography, age, and food. Furthermore, finding reliable microbiological biomarkers is made more difficult by the variety of DN symptoms.

In summary, the gut microbiome is both a contributor to the development of diabetic neuropathy and a biomarker for it. Microbiome-targeted therapies may change the progression of the disease, but gaps remain in our understanding of causality and stability. To make progress, multi-omic research and a multidisciplinary clinical approach are needed to improve outcomes

## 6 Conclusion

A potential conceptual shift in the study of diabetic neuropathy is the integration of microbial science. Via the gut-brain-peripheral nerve axis, we have evidence that dysbiosis of the gut causes systemic inflammation, metabolic dysfunction, and nerve injury. While treatments that modify the microbiota might have a disease-modifying effect, multiomic studies offer substantial evidence for the use of microbiome-based biomarkers for the early diagnosis of DN. To confirm these results and enable their therapeutic use, however, larger, longer-term, and standardized investigations are required. To improve studies and personalized therapy of DN, a multidisciplinary strategy integrating endocrinology, neurology, microbiology, and nutrition is required.
